# Radiocarbon as a Novel Tracer of Extra-Antarctic Feeding in Southern Hemisphere Humpback Whales

**DOI:** 10.1038/s41598-017-04698-2

**Published:** 2017-06-29

**Authors:** Pascale Eisenmann, Brian Fry, Debashish Mazumder, Geraldine Jacobsen, Carlysle Sian Holyoake, Douglas Coughran, Susan Bengtson Nash

**Affiliations:** 1 Griffith University, Environmental Futures Research Institute (EFRI), Southern Ocean Persistent Organic Pollutants (SOPOPP), Brisbane, QLD 4111 Australia; 2 Griffith University, Australian Rivers Institute (ARI), Brisbane, QLD 4111 Australia; 3 Australian Nuclear Science and Technology Organisation (ANSTO), Lucas Heights, NSW 2234 Australia; 4 Murdoch University, Perth, WA 6150 Australia; 5 Department of Parks and Wildlife, Kensington, WA 6151 Australia

## Abstract

Bulk stable isotope analysis provides information regarding food web interactions, and has been applied to several cetacean species for the study of migration ecology. One limitation in bulk stable isotope analysis arises when a species, such as Southern hemisphere humpback whales, utilises geographically distinct food webs with differing isotopic baselines. Migrations to areas with different baselines can result in isotopic changes that mimic changes in feeding relations, leading to ambiguous food web interpretations. Here, we demonstrate the novel application of radiocarbon measurement for the resolution of such ambiguities. Radiocarbon was measured in baleen plates from humpback whales stranded in Australia between 2007 and 2013, and in skin samples collected in Australia and Antarctica from stranded and free-ranging animals. Radiocarbon measurements showed lower values for Southern Ocean feeding than for extra-Antarctic feeding in Australian waters. While the whales mostly relied on Antarctic-derived energy stores during their annual migration, there was some evidence of feeding within temperate zone waters in some individuals. This work, to our knowledge, provides the first definitive biochemical evidence for supplementary feeding by southern hemisphere humpback whales within temperate waters during migration. Further, the work contributes a powerful new tool (radiocarbon) for tracing source regions and geographical feeding.

## Introduction

## Ecological tracers: isotopes

Carbon and nitrogen stable isotope measurements (δ^13^C and δ^15^N values) are routinely used to study food webs interactions in both marine and terrestrial environments. Isotope values are derived from diet and are recorded in animal tissues^1, 2^. In general, stable isotope (SI) values are excellent tools for the interpretation of changes in feeding locations, catabolic reactions, and for assigning the trophic level of a species^1–3^. Problematically, particularly in the case of migratory species, geographical^4, 5^ and temporal^6, 7^ isotopic gradients must be accounted for prior to these interpretations. For migrants, tissue turn-over dynamics and trophic fractionation can complicate the identification of correct feeding locations and prey intake proportions^8, 9^. Additionally, isotopic pools with differing origins, such as body reserves, muscle protein, and direct food intake, can have non-equal contributions to tissue formation^3, 10^. These problems combine in the isotopic records from Antarctic migratory species that travel between geographically distinct food webs to the North and South of the Antarctic Circumpolar Current. In such migrants, energy reserves acquired from distinct feeding locations are combined, resulting in the bulk δ^13^C and δ^15^N values of long-term stores being effectively averaged. As the two different isotopic pools merge and equilibrate, using bulk SI to isolate separate feeding events is rendered more difficult.

Radiocarbon (^14^C) is expected to be particularly useful in these scenarios for two reasons. Firstly, in determining Δ^14^C, the ^14^C measurement is corrected for isotopic fractionation as well as the amount of decay between time of death and time of measurement^11^, thus allowing comparisons with other Δ^14^C results. As a result, unlike δ^13^C and δ^15^N values, the Δ^14^C values obtained are a direct reflection of a specific source, and are not affected by trophic level, productivity or the composition of the prey. Secondly, while most ^14^C forms naturally in the upper atmosphere through interactions of cosmic ray neutrons with nitrogen, ^14^C was also artificially released during the 1950’s and 1960’s thermonuclear tests. This rapid increase in atmospheric ^14^C content, known as the “bomb-peak”, has slowly been absorbed by the biosphere and surface oceans, transferring the anomalously high Δ^14^C values to surface water around the globe^12^. These surface Δ^14^C values vary both regionally and temporally^13, 14^. Because ^14^C slowly decays over time (with a half-life of 5730 years), global oceanic cycling causes deep water, isolated from atmospheric exchange for decades to centuries, to be depleted in ^14^C relative to surface waters^15, 16^. The unique overturning cells of the Southern Ocean cause older, deep water from the surrounding oceans to upwell south of the Polar Front Zone^17^. Consequently, Southern Ocean surface waters are more depleted in radiocarbon than temperate surface waters^12, 18^. Because these oceanic signals are transferred through the food web, marine wildlife restricted in their foraging to regions south of the Polar Front Zone^19–21^ also have lower Δ^14^C values than populations whose foraging ranges extend into temperate waters^13, 22^.

Radiocarbon is thus a potentially valuable tracer for studying which source regions are utilised for feeding by Southern Ocean migratory fauna. Improved understanding of the importance of feeding in the Southern Ocean should significantly contribute to our understanding of the diverse environmental challenges faced in this region. For example, Polar Skua (*Stercorarius maccormicki*) are known to utilise both Antarctic summer feeding and breeding grounds, as well as temperate northern latitude feeding grounds contaminated by pollutants^23–25^. Their role as biological “vectors” of contamination input into the Antarctic could benefit from the potential advantages of using radiocarbon as a tracer. Similarly, a recent increase in observations of feeding outside of the Antarctic (extra-Antarctic feeding) by Southern Hemisphere Humpback whales (*Megaptera novaeangliae*, SHHWs) may signal a change to the abundance or availability of their primary food source, Antarctic krill (*Euphausia superba*).

Antarctic krill is a species posed as particularly vulnerable to climate change and sea-ice loss^26–28^. Since SHHWs are understood to be Antarctic Krill obligates^29, 30^, instances of feeding outside of the Antarctic are anomalous and need to be investigated. Previous work by the author team on baleen bulk SI profiles baleen revealed unexpected heterogeneity in recent feeding events undertaken by stranded humpback whales from two Australian populations^31^. While the isotopic profiles were generally consistent with feeding on prey other than Antarctic krill, intrinsic characteristics of bulk SI analysis created uncertainties in the explicit description of those events^31^. In particular, clear differentiation between Antarctic feeding at a higher trophic level than Antarctic krill, and feeding outside of the Antarctic was not possible using only the bulk SI approach. The ability to robustly differentiate between extra-Antarctic feeding behaviours^29, 31–33^ as either anomalous and opportunistic events, or as a widespread, routine feeding behaviour remains a key challenge in the study of current and changing humpback whale energetic health and ecology.

In order to provide further context to ambiguous feeding signals in bulk SI profiles of humpback whale baleen plates, a sub-sample of the plates analysed for bulk SI were selected for radiocarbon measurement. Skin samples were additionally selected to serve as short-term records of recent feeding. Radiocarbon analyses are generally expensive, so only a limited number of samples are typically analysed. We selected the samples to test the hypothesis that Δ^14^C would more robustly indicate geographical feeding areas than δ^13^C alone, and would likely co-vary with the traditional geographical feeding marker δ^13^C. Thus, it was expected that high values of both δ^13^C and Δ^14^C would indicate extensive feeding within temperate waters, while low values would indicate feeding within Antarctic waters. As with δ^13^C values, it is not expected that Δ^14^C will completely turn over to extra-Antarctic values as a consequence of supplementary feeding, but rather will fall on a spectrum between pure Antarctic feeding and pure extra-Antarctic feeding. Discrepancies from this pattern, such as a decoupling between δ^13^C and Δ^14^C, might occur if feeding has taken place within Antarctic upwelling waters with differing productivities^34^.

## Results and Discussion

### Radiocarbon records in baleen plates

**Figure 1:**
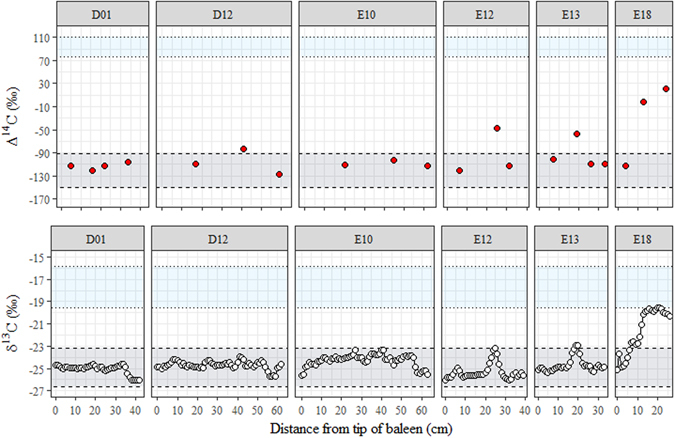
Temporal representation of baleen Δ^14^C and δ^13^C in baleen plates. This figure has been modified from Eisenmann, *et al*.^31^. Each horizontal zone is created using location-specific isotopic estimates consistent with prey values and trophic fractionation (if applicable). The whale isotopic data plots within the zone appropriate to provisioning location. Isotopic zones: Australian prey – merged (dotted line), Antarctic prey (dashed line).

Overall, baleen radiocarbon values grouped closely within the zone corresponding to feeding within Antarctica (Fig. 1), with few samples plotting outside this range. These results hereby support the SI findings reported previously, with most of the baleen observations belonging to the category described as “Classical Feeders” (i.e. high-fidelity feeding on Antarctic Krill, interspersed with extensive fasting^31^).

**Figure 2:**
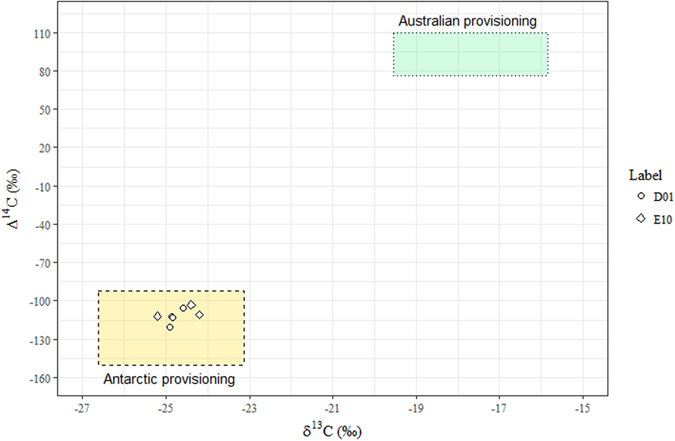
Bivariate representation of baleen Δ^14^C and δ^13^C in classical feeders. Coloured zones represent isotopic values for the Antarctic feeding grounds (gold, dashed line), and Australian feeding grounds (green, dotted line).

Isotopic values for animals D01 and E10 were consistently located within the Antarctic feeding zone, with radiocarbon values constrained between −90‰ and −150‰ (Fig. 1, Fig. 2). This confirms the previous classification of these whales as Classical Feeders^31^.

**Figure 3:**
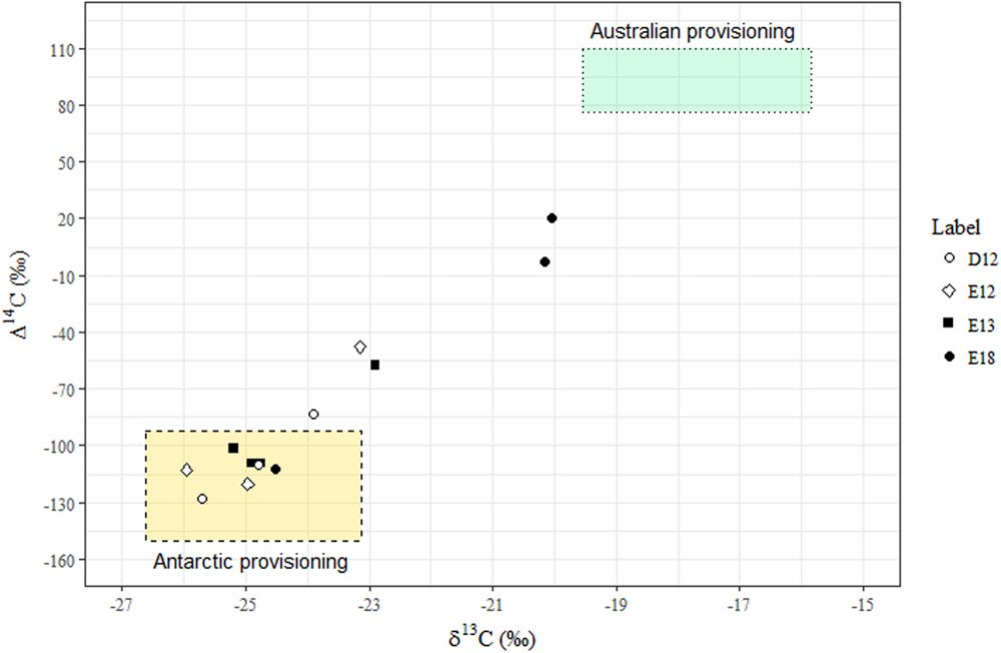
Bivariate representation of baleen Δ^14^C and δ^13^C in whales engaging in non-classical feeding. Coloured zones represent isotopic values for the modern Antarctic feeding grounds (gold, dashed line), and Australian feeding grounds (green, dotted line).

By contrast, Δ^14^C values for whale D12, previously identified as a Classical Feeder^31^, plotted more similarly to whales E12 and E13, characterised as Supplementary Feeders^31^ (Fig. 1, Fig. 3). Δ^14^C values for these three animals were only partially located within the modern Antarctic zone, with a singular point outside the range for each individual (Fig. 1, Fig. 3). The respective trendlines between the centres of the Antarctic and Australian feeding zones are approximately Δ^14^C = 31* δ^13^C + 660. The general trendline for whales with radiocarbon values outside of the Antarctic zone was Δ^14^C = 25* δ^13^C + 500, placing Supplementary Feeders D12, E12 and E13 on the same pathway towards the Australian feeding zone (Fig. 3). A combination of Antarctic and extra-Antarctic feeding was therefore consistent with both the SI and radiocarbon patterns observed for whales E12 and E13, conclusively demonstrating extra-Antarctic feeding during migration in the recent pre-stranding life history of sampled SHHWs. The unexpected D12 results indicate that radiocarbon analysis was more sensitive than bulk δ^13^C analysis in detecting supplementary feeding events. While the data point was only outside our literature-defined radiocarbon boundary by ~10‰ (Table 1), this correlation between δ^13^C and Δ^14^C suggested that D12 also engaged in recent supplementary feeding during migration, although the quantities of non-Antarctic food consumed were not large enough to strongly change the bulk SI toward Australian values.

**Table 1:** Δ^14^C and δ^13^C (‰) for the samples used in this study. A – Baleen plate samples. B – Skin samples.

A	Distance from tip (cm)	δ^13^C (‰)	pMC (%)	Error	Δ^14^C (‰)	Error	ANSTO ID
D01	5	−24.9	89.4	0.3	−112.4	2.8	OZS180
	16	−24.9	88.6	0.2	−120.5	2.3	OZS181
	22	−24.9	89.3	0.2	−112.9	2.3	OZS182
	34	−24.6	90.1	0.2	−105.5	2.2	OZS183
E10	20	−24.2	89.6	0.3	−111.0	3.0	OZT109
	45	−24.4	90.4	0.3	−103.0	3.0	OZT110
	62	−25.2	89.5	0.3	−112.0	3.0	OZT111
E12	6	−25.0	88.7	0.3	−120.2	2.9	OZS530
	25	−23.2	96.0	0.3	−47.8	3.0	OZS531
	31	−26.0	89.4	0.3	−112.7	2.8	OZS532
E13	7	−25.2	90.6	0.3	−101.6	2.7	OZS533
	19	−22.9	95.0	0.4	−57.6	3.8	OZS534
	26	−24.8	89.8	0.3	−109.2	3.1	OZS535
	33	−24.9	89.8	0.3	−109.2	3.0	OZS536
E18	4	−24.5	89.5	0.3	−112.3	3.0	OZS527
	13	−20.2	100.5	0.5	−2.5	4.6	OZS528
	24	−20.0	102.8	0.3	20.2	2.9	OZS529
D12	17	−24.8	89.7	0.3	−110.0	3.0	OZT106
	41	−23.9	92.5	0.3	−83.0	3.0	OZT107
	59	−25.7	87.9	0.3	−128.0	3.0	OZT108
**B**	**Collection Location**	**δ** ^**13**^ **C (‰)**	**pMC (%)**	**Error**	**Δ** ^**14**^ **C (‰)**	**Error**	**ANSTO ID**
D06-S	West Australia	−25.1	90.6	0.2	−101.0	2.0	OZT112
E08-S	East Australia	−27.3	89.3	0.2	−114.0	2.0	OZT113
E14-S	East Australia	−29.6	91.9	0.2	−88.0	2.0	OZT114
1A13	Antarctica	/	90.7	0.3	−101.0	3.0	OZT115
7A13	Antarctica	−24.1	89.8	0.3	−109.0	2.0	OZT116
13s13	East Australia	−27.3	89.7	0.2	−110.0	2.0	OZT117

Radiocarbon data is also presented in percent Modern Carbon (pMC); the errors shown correspond to 1 standard deviation. Low numbers for the distance from tip in baleen correspond to older isotope records, i.e. 0 is the oldest part of the baleen while larger numbers are more recent in time.

Whale E18 (Fig. 1, Fig. 3) was previously determined to be an example of a whale that remained within temperate waters in the years preceding death, with an enrichment in δ^13^C and δ^15^N over time consistent with a directional change from Antarctic feeding to Australian feeding^31^. Stable isotope profiles of this sort are best explained by long-term diet changes, such as partial migration in the case of the SHHWs, where the individual stays in temperate waters instead of returning to the Antarctic feeding grounds for a complete migration cycle^35^. This SI interpretation was further supported by the fact that whale E18 stranded in December 2012, the height of the Austral summer, when the whales are expected to be feeding in Antarctica. Radiocarbon values for this whale were consistent with its tentative classification as a Temperate Zone feeder^31^ (Fig. 1, Fig. 3), reaching values close to those of Australian prey species.

The combination of δ^13^C and Δ^14^C provided a robust, more powerful way to detect supplementary feeding in SHHW baleen plates than δ^13^C or δ^15^N alone. While several plausible causation factors remained following traditional SI analysis^31^, complementary Δ^14^C analysis definitively showed the high δ^13^C values previously measured in some SHHWs baleen plates were indicative of feeding in two different regions.

### Radiocarbon records in skin

**Figure 4:**
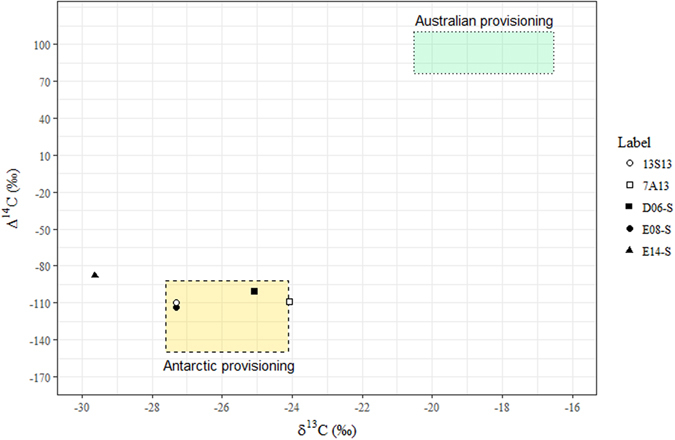
Bivariate representation of skin Δ^14^C and δ^13^C. Coloured zones represent isotopic values for the Antarctic feeding grounds (gold, dashed line), and Australian feeding grounds (green, dotted line). Biopsied samples are in white, stranded individuals in black. 1A13 is not shown here due to missing δ^13^C data.

Unlike baleen plates, skin turn-over is relatively fast, and expected to fully shift to “new isotope values” in a matter of weeks. Thus, isotopic values in sampled skin should reflect Antarctic provisioning in most cases, except for recent supplementary feeders. Four out of five skin radiocarbon measurements plotted within the Antarctic feeding zone, with the fifth (E14-S) plotting close by (Fig. 4, dashed-zone). Skin samples from all three biopsied whales 13S13, 1A13, and 7A13, and from stranded whales D06 and E08 fit the radiocarbon estimates for Antarctic provisioning (Fig. 4).

Baleen plates from stranded whales D06, E08 and E14 were previously analysed for SI. Whale E08 was identified as a classical feeder, while whale E14 was identified as a supplementary feeder^31^. In addition, baleen isotope records from neonate D06 indicated that maternal provisioning originated exclusively from Antarctic feeding grounds (Supplementary Table 2). Radiocarbon measurements for these three whales confirmed the classification that resulted from SI analysis of their baleen plates (Fig. 4). The skin sample from whale E14, however, was slightly outside the Antarctic radiocarbon range (Fig. 4). While it cannot be proven retrospectively, the particularly low δ^13^C values for whale E14 may, for example, be explained by an incomplete lipid extraction prior to SI analysis, particularly as they do not fit the comparatively high Δ^14^C values, outside of the Antarctic feeding range (Fig. 4). Productivity variations within Antarctic food webs may also potentially alter δ^13^C (but not Δ^14^C), with a wide δ^13^C Antarctic range already documented in SHHW baleen^31^. Productivity models that focus on isotope fractionation during carbon fixation predict lower δ^13^C values for phytoplankton areas with low productivity, and higher δ^13^C values for areas with high productivity^36, 37^. These values would be passed up the food web to consumers such as whales. Further exploration of this potential productivity response with a combination of radiocarbon and stable carbon isotopes could be important for better understanding ecosystem productivity in a time of accelerated Antarctic climate change.

As expected by the fractionation-correction applied during measurement, Δ^14^C did not appear to be affected by metabolism or fasting in humpback whale tissues. As such, Δ^14^C is in many ways a superior tracer to δ^13^C or δ^15^N for Antarctic migrating marine wildlife, since variations were directly linked to feeding in different bodies of water and not complicated by metabolic changes. This was especially visible in the skin, where samples collected in Australian and Antarctic waters showed exclusively Antarctic radiocarbon values, while the δ^13^C fluctuated more strongly.

In conclusion, we showed that the use of radiocarbon in Antarctic migratory biota provided an excellent, novel tracer contributing critical resolution to the traditional δ^13^C and δ^15^N tracer measurements. In this study, its use allowed us to definitively separate mixed source feeding of SH humpback whales.

## Methods

### Ethical Statement

Baleen plates and skin were collected from animals stranded or biopsied in Australia between 2007 and 2013. All but animal D06, a neonate, were classified as adults at the time of collection. Collection and sample details are given in Table S1. These animals belong to both the east (E1) and west (D) coast migrating populations^38^. Baleen and skin samples obtained from necropsied whales were preserved in scientific and museum collections until requested for analyses. In the case of the Southern Ocean Persistent Organic Pollutant Program (SOPOPP) collection, necropsy samples and biopsies were obtained under Scientific Purposes permits WISP14251214 and WISP14257414, granted by the QLD Department of Environment and Heritage Protection and Animal Ethics permit ENV1710AEC granted by the Griffith University Animal Ethics Committee, in accordance with the approved methods outlined in the permits.

### Sampling and storage

Skin and baleen plates were collected from Australian stranded animals between 2007 and 2012. A further three skin biopsies were collected from free-ranging Antarctic animals during the 2012–2013 austral summer through the Southern Ocean Research Partnership Blue whale voyage. All but one of the samples were kept frozen after collection. The exception to this rule were samples from whale D01. Because the carcass from D01 was buried during decomposition, plates and tissues collected had degraded. As a result, the possible presence of soil materials on plates D01 was a concern for radiocarbon contamination.

Biopsied animals are in the form “1X11”, where the first number gives the biopsy order for the animal and the last two numbers are the year of collection. The first number restarts each season. A refers to animals sampled in Antarctica. Stranded animal IDs are in the form of “X11”, with E or D referring to the breeding population. The two numbers refer to the collection order, and are not related to sampling date.

### Sample selection

No assumptions were made regarding the individual whale’s feeding behaviour prior to δ^13^C and δ^15^N SIA. For bulk SI analysis, the full length of each individual baleen plate was sampled and analysed at 1cm increments. Such increments have been showed to represent approximately 1 month of growth in baleen whales^31, 39^, providing an isotopic temporal record of feeding events. δ^13^C and δ^15^N baleen profiles revealed heterogeneous feeding strategies in the recent feeding history of stranded SHHWs^31^. While some individuals conformed to the classical model of high fidelity Antarctic Krill feeding, individual SI patterns fell on a broad spectrum spanning from high-fidelity Antarctic krill diet to complete reliance on temperate prey items. For the purpose of further analysis and discussion, the observed bulk SI profiles were divided into three categories: Classical Feeders (high-fidelity feeding on Antarctic krill interspersed with migratory fasting), Supplemental Feeders (defined here as extraneous, discrete feeding events not conforming to the classical feeding model), and Temperate Zone Feeders^31^. Whales D01, E10, D12, E08 and D06 were identified as belonging to the classically feeding category, with SI records consistent with low trophic level Antarctic provisioning^31^. In the case of neonate D06, which would not have ever fed on its own, the baleen profile indicated Antarctic-sourced maternal provisioning. As a result, D06 was classified as a classical feeder, although indirectly. Animal E18 was classed as a temperate zone feeder, with baleen records indicating a long-term, directional shift to Australian feeding. Finally, whales E12, E13, and E14 were classified as exhibiting moderate supplementary feeding at a higher trophic level and/or outside Antarctic feeding grounds^31^.

Radiocarbon sampling location on baleen plates from whales D01, E10, D12, E18, E12 and E13 was selected visually according to the SI profiles^31^ so as to provide the best coverage of interesting features in the SI records. For example, peaks and through in the profiles were selected as possible locations for shifts in feeding behaviour, and corresponding shifts in radiocarbon values. Skin samples from whales E08, D06 and E14 were selected to identify whether short-term radiocarbon values were consistent with long-term plate SI records. Two Antarctic skin biopsies were also included in the radiocarbon analyses, as well as a skin biopsy from an adult male obtained during its southward migration along the East coast of Australia in 2013. The sample from Antarctic biopsy 1A13 was insufficient to conduct subsequent bulk SI analyses.

Information regarding sample codes, feeding category and sample sources are provided in Table S1, while the full bulk SI values for each baleen plate are reported in Table S2. A subsample of values for notches analysed for both SI and radiocarbon are given in Table 1. Complete adult baleen profiles can be found in Eisenmann, *et al*.^31^, but δ^13^C profiles for the baleen analysed here are visible in Fig. 1.

### Radiocarbon analysis

Skin samples were lipid extracted following a modified Bligh and Dyers chloroform extraction^40^, as used in previous work^41, 42^. Samples were extracted overnight using a methanol/dichloromethane (DCM)/water (MeOH/CH_2_Cl_2_/H_2_O) extraction (2:1:0.8 v/v/v). After addition of DCM/water (1:1:0.9 v/v/v), samples were left to partition into the aqueous and DCM phases for another day. The extracted tissue was collected and air-dried. All baleen plates were washed in deionised water, and in a 2:1 chloroform:methanol solution prior to analysis to ensure all contaminants and residual lipids were removed. Material for radiocarbon analysis was collected from the cleaned baleen plates using a rotary blade. The powdered material from D01 was further washed with 0.1M NaOH at 60 °C for 1 hour to remove soil contaminants.

Powdered baleen and skin samples were then pre-treated with 2M HCl at 60 °C for 1 hour to remove carbonates. The pre-treated material was combusted to CO_2_ using the sealed tube technique^43^ and then converted to graphite using the H_2_/Fe reduction^43^. AMS ^14^C measurements were carried out using the STAR 2MV Tandetron at the Australian Nuclear Science and Technology Organisation (ANSTO) facility^44^. Radiocarbon results are reported in Table 1 as percent Modern Carbon (pMC) and Δ^14^C (‰) as per Stuiver and Polach^11^. Previously measured δ^13^C values for all points sampled for radiocarbon analysis are also reported in Table 1 ^31^. Unfortunately, one of the Antarctic biopsies lacked sufficient material to obtain both δ^13^C/δ^15^N and Δ^14^C values.

### Location-specific isotopic range estimates

Location-specific isotope range estimates were necessary to infer the feeding location of the whales depending on prey items consumed. Location-specific estimates were calculated for both δ^13^C and Δ^14^C. The estimates were then used to compare whether the measured radiocarbon values matched the δ^13^C–inferred geographical location of feeding. For example, a whale with measured δ^13^C values falling within the Antarctic-specific δ^13^C range estimate is expected to have radiocarbon values falling within the Antarctic-specific Δ^14^C range estimate.

### δ^13^C range estimates

Location-specific δ^13^C ranges were defined using Antarctic and Australian prey samples reported in the literature^2, 45–49^, and adjusted for trophic fractionation using a trophic estimate (TEF) appropriate for baleen tissue^31^ (Equation 1). The adjusted estimates are calculated as follow:

1 $${\delta }^{13}{C}_{x}=TE{F}_{x}+{\delta }^{13}{C}_{p}\pm S{D}_{p}$$

where x = sample type, p = prey, TEF is the trophic enrichment factor and SD is the standard deviation^31^.

The isotopic estimates include standard errors to account for temporal, taxonomic and sub-regional variability in bulk isotopes. Because it is unknown which prey type is selected by the SHHWs once they reach Australian waters, the Australian-specific range estimates were created using the outside boundaries of two previously calculated estimates. Australian krill and Australian fish estimates were therefore combined to provide one large isotopic “zone” encompassing exclusive and mixed trophic level feeding events. Similar predictions were calculated for skin tissue using the same prey data, and appropriate skin TEFs^50^. These are presented in Table 2.

**Table 2:** Expected isotopic value depending on prey choice and tissue type.

Location/Prey combinations	δ^13^C TEF			References
	Baleen (+2.26‰)	Skin (+1.28‰)	±SD	
Whales consuming 100% Antarctic Krill	−24.9	−25.9	1.7	Cherel^2^, Wada, *et al*.^46^, Hall-Aspland, *et al*.^47^, Hodum and Hobson^48^
Whales consuming 100% Australian Krill	−17.7	−18.4	0.4	Harris, *et al*.^49^
Whales consuming 100% Australian fish	−16.5	−17.4	0.9	Calculated from Davenport and Bax^45^
Whales on a mixture of Australian prey species	−18.4	−16.5	0.9	Calculated from Harris, *et al*.^49^, Davenport and Bax^45^

Antarctic krill samples originate from within Antarctic Area IV and V (which correspond to the most likely feeding grounds for these SHHWs populations), and were collected between 1982 and 2002. Multiple species of Australian krill were collected off the East Australian coast in 2010, and multiple species of Australian pelagic fishes (both secondary and tertiary consumers) were sampled in the Bass straits, off the South-east Australian coast, between 1993 and 1996. Australian prey items were averaged, because any of the species potentially could be a prey item. TEF refers to the trophic enrichment factor used to correct the prey values to the trophic level of the whales.

### Δ^14^C range estimates

Location-specific radiocarbon estimates (including error ranges) were similarly calculated using values reported in the literature (Table 3). Since radiocarbon is not influenced by trophic level, no trophic correction was applied.

**Table 3:** Reported Δ^14^C values for water and marine wildlife in Antarctic and Australia.

Location	Radiocarbon value (Species, Year Collected)		References
	Pre-Bomb Δ^14^C (‰)	Post Bomb Δ^14^C (‰)	
Antarctic feeding	−170 to −140 (Molluscs, 1917–1940), −175 to −117 (Whalebone, pre-nuclear), −149 (Penguin, 1912)	−110 (Molluscs, 1995), −96 to −92 (Krill, 1980), −150 to −111 (Algae, 1978 to 1980)	Berkman and Forman^19^, Gordon and Harkness^20^, Michel and Druffel^21^, Adamson and Pickard^51^, Zhang, *et al*.^52^, Stuiver, *et al*.^53^
SH Temperate water feeding	−50 to −40 (Shells, 1853–1914), −52 (Coral, 1950–1956), −48 (Fish, 1918)	110 (Coral, 1991), 100 (Coral, 1996), 76.5 (Fish, 1990)	Guilderson, *et al*.^22^, Delibrias^54^, Kalish^55^

Reported values for pre- and post-bomb radiocarbon present in various local Antarctic and southern hemisphere temperate surface waters organisms.

When plotted in 2D space, the estimates form location-specific isotopic zones where whale samples are expected to fall depending on the type of provisioning.

## Electronic supplementary material

Supplementary data
