# Supplementary material for: Radiocarbon as a Novel Tracer of Extra-Antarctic Feeding in Southern Hemisphere Humpback Whales
Source: Sci Rep. 2017 Jun 29;7:4366. doi: 10.1038/s41598-017-04698-2 (PMC5491506; doi:10.1038/s41598-017-04698-2)
Supplement: Supplementary file 1 — Supplementary data [file 41598_2017_4698_MOESM1_ESM.pdf]

Radiocarbon as a Novel Tracer of Extra-Antarctic Feeding in Southern Hemisphere Humpback Whales

Pascale Eisenmann[1,*], Brian Fry[2], Debashish Mazumder[3], Geraldine Jacobsen[3], Carlysle Sian Holyoake[4], Douglas Coughran[5], Susan Bengtson Nash[1]

[1] Griffith University, Environmental Futures Research Institute (EFRI), Southern Ocean Persistent Organic Pollutants (SOPOPP), Brisbane QLD 4111, Australia

[2] Griffith University, Australian Rivers Institute (ARI), Brisbane QLD 4111, Australia

[3] Australian Nuclear Science and Technology Organisation (ANSTO), Lucas Heights NSW 2234, Australia

[4] Murdoch University, Perth WA 6150, Australia

[5] Department of Parks and Wildlife, Kensington WA 6151, Australia

* Corresponding Author: pascale.eisenmann@griffithuni.edu.au

Supplementary Information

Table S1: Details of the individual animals used in this study, listed under their bulk isotope-assigned feeding category

ID	***Date Collected***	***Tissue type***	***Collection location***	***Collector / Curator***	***ANSTO ID***	***Sex***	***Size in meter***
**Classical Feeding**							
D01*	30/07/07	Baleen	Hillary's Boat Harbour (WA)	Murdoch University[1]	OZS184-187	/	12
E10*	01/10/11	Baleen	Main Beach, North Stradbroke Is. (QLD)	SOPOPP[2]	OZT109-111	M	14
D12*	18/08/13	Baleen	(WA)	Murdoch University[1]	OZT106-108	F	17
E08-S*	07/06/10	Skin	Tallebudgera (QLD)	SOPOPP[2]	OZT113	/	7.9
D06-S	25/08/11	Skin	Jurien Bay (WA)	Murdoch University[1]	OZT112	M	4.35
**Supplementary Feeding**							
E12*	06/11/11	Baleen	Tasmanian Peninsula (TAS)	DPIPWE [3]	OZS530-532	M	10.6
E13*	07/11/11	Baleen	Bruny Island (TAS)	DPIPWE [3]	OZS533-536	M	8.6
E14-S*	05/06/12	Skin	Fraser Is., South Eli beach (QLD)	Queensland Museum[5]	OZT114	M	8.02
**Partial Migration**							
E18*	Dec 2012	Baleen	Venus Bay (VIC)	Museum Victoria[6]	OZS527-529	/	/
**No Classification**							
1A13	12/2012 - 01/2013	Skin	Antarctica	AAD[4]	OZT115	F	Adult (>6m)
7A13	12/2012 - 01/2013	Skin	Antarctica	AAD[4]	OZT116	M	Adult (>6m)
13S13	22/09/13	Skin	North Stradbroke Is. (QLD)	SOPOPP[2]	OZT117	M	Adult (>6m)

Date and location of stranding are reported here, as well as the identity of the collector. When known, sex was determined either through visual inspection during sample collection or through DNA-sexing of matching blubber sample. E, D and A in the sample ID refer to the sampled population, where E and D respectively represent the Eastern Australian (E1) and Western Australian (D) migrating breeding population, and A represents individual samples on the Antarctic feeding grounds. "-S" in the sample ID indicates that radiocarbon was measured in a skin sample and not a baleen plate. Individual adult whales with baleen SI profiles previously published[23] are indicated with and asterisk. All animals >6m in length are considered adults for the purpose of this study. [1]Carly Holyoake, Murdoch University WA. [2]Southern Ocean Persistent Organic Pollutant Program (SOPOPP), Griffith University QLD. [3]Tasmanian Department of Primary Industries, Parks, Water and Environment (DPIPWE), TAS. [4]Southern Ocean Research Partnership (SORP) 2012 Voyage, Australian Antarctic Division, TAS. [5]Queensland Museum, Brisbane QLD. [6]Museum Victoria, Carlton

Whale ID	Sampling notch #	δ^{13}C	δ^{15}N	Whale ID	Sampling notch #	δ^{13}C	δ^{15}N
E01	0	-22.9	7.2	E13	0	-25.2	7.2
E01	1	-23.1	7.3	E13	1	-24.9	6.9
E01	2	-23.2	7.4	E13	2	-24.9	7.1
E01	3	-23.3	7.5	E13	3	-25.1	6.7
E01	4	-23.3	7.6	E13	4	-25.3	6.9
E01	5	-23.2	7.5	E13	5	-25.4	7.1
E01	6	-23.2	7.6	E13	6	-25.2	7.1
E01	7	-22.8	7.7	E13	7	-25.2	7.2
E01	8	-22.7	8.0	E13	8	-25.0	7.4
E01	9	-22.5	8.1	E13	9	-24.9	7.9
E01	10	-22.2	8.3	E13	10	-24.9	7.8
E01	11	-22.0	8.5	E13	11	-24.9	7.8
E01	12	-21.5	8.6	E13	12	-24.9	7.8
E01	13	-21.4	8.8	E13	13	-24.9	7.8
E01	14	-21.4	9.0	E13	14	-24.9	7.8
E01	15	-21.9	8.6	E13	15	-24.8	7.8
E01	16	-22.3	8.5	E13	16	-24.4	7.9
E01	17	-22.0	8.3	E13	17	-23.6	8.2
E01	18	-21.9	8.2	E13	18	-23.0	8.4
E01	19	-21.8	7.8	E13	19	-22.9	9.2
E01	20	-21.4	7.6	E13	20	-22.9	8.9
E01	21	-20.5	7.7	E13	21	-23.7	8.1
E01	22	-20.1	7.9	E13	22	-24.5	7.1
E01	23	-19.3	8.3	E13	23	-24.8	6.0
E01	24	-18.7	8.5	E13	24	-24.7	5.8
E01	25	-18.3	9.6	E13	25	-24.6	6.0
E01	26	-18.4	9.9	E13	26	-24.8	6.5
E01	27	-18.5	10.6	E13	27	-25.2	6.8
E01	28	-18.5	10.5	E13	28	-25.3	6.8
E01	29	-18.4	10.5	E13	29	-24.8	6.8
E01	30	-18.3	10.1	E13	30	-24.7	7.3
E01	31	-17.9	9.9	E13	31	-24.8	7.4
E01	32	-17.8	10.4	E13	32	-24.9	7.3
E01	33	-17.9	10.4	E13	33	-24.9	7.2
E01	34	-18.2	10.6	E18	0	-25.1	7.6
E08	0	-26.0	6.0	E18	1	-25.0	7.8
E08	1	-26.0	6.1	E18	2	-24.9	8.0
E08	2	-26.0	6.3	E18	3	-24.8	8.2
E08	3	-26.1	6.1	E18	4	-24.5	8.5
E08	4	-26.1	6.2	E18	5	-24.0	8.9
E08	5	-26.0	6.1	E18	6	-23.4	9.1
E08	6	-25.9	6.4	E18	7	-22.7	9.5
E08	7	-25.8	6.6	E18	8	-22.6	9.7

E08	8	-25.5	6.4	E18	9	-22.8	9.9
E08	9	-25.1	6.5	E18	10	-22.8	9.4
E08	10	-24.9	6.3	E18	11	-22.2	9.4
E08	11	-25.0	6.2	E18	12	-21.1	9.4
E08	12	-25.2	6.7	E18	13	-20.2	9.6
E08	13	-25.4	6.5	E18	14	-19.9	9.7
E08	14	-25.7	6.7	E18	15	-19.8	10.1
E08	15	-25.9	6.8	E18	16	-19.7	10.3
E08	16	-25.6	6.4	E18	17	-19.8	10.5
E08	17	-25.5	6.7	E18	18	-19.9	10.6
E10	0	-25.6	4.8	E18	19	-19.7	10.6
E10	1	-25.5	5.5	E18	20	-19.6	10.5
E10	2	-24.9	6.1	E18	21	-19.5	11.1
E10	3	-24.8	6.1	E18	22	-19.6	11.3
E10	4	-24.4	6.0	E18	23	-20.0	11.5
E10	5	-24.6	5.9	E18	24	-20.0	11.5
E10	6	-24.6	6.2	E18	25	-20.2	11.7
E10	7	-24.7	6.1	E18	26	-20.3	11.6
E10	8	-24.3	5.9	D01	0	-24.7	5.9
E10	9	-24.3	5.9	D01	1	-24.7	5.4
E10	10	-24.3	6.1	D01	2	-24.8	5.6
E10	11	-24.1	5.8	D01	3	-25.0	5.0
E10	12	-24.1	6.0	D01	4	-25.0	4.9
E10	13	-24.2	5.0	D01	5	-24.9	5.3
E10	14	-24.4	5.1	D01	6	-24.9	5.3
E10	15	-24.1	5.2	D01	7	-25.0	5.1
E10	16	-24.1	5.3	D01	8	-25.0	5.3
E10	17	-24.0	6.0	D01	9	-24.9	5.9
E10	18	-24.2	6.2	D01	10	-24.9	5.9
E10	19	-24.1	6.3	D01	11	-25.0	6.2
E10	20	-24.2	6.3	D01	12	-24.8	6.0
E10	21	-24.1	5.7	D01	13	-24.9	6.1
E10	22	-24.0	6.1	D01	14	-25.1	6.2
E10	23	-24.0	5.8	D01	15	-24.9	6.2
E10	24	-24.0	6.5	D01	16	-24.9	2.8
E10	25	-23.9	5.8	D01	17	-24.8	6.3
E10	26	-23.8	6.4	D01	18	-24.7	6.1
E10	27	-23.3	5.6	D01	19	-24.7	6.0
E10	28	-24.0	5.1	D01	20	-24.8	5.5
E10	29	-24.1	4.7	D01	21	-25.2	5.1
E10	30	-24.0	4.9	D01	22	-24.9	4.7
E10	31	-24.3	4.9	D01	23	-25.0	4.7
E10	32	-24.4	4.6	D01	24	-25.0	4.7
E10	33	-24.3	5.1	D01	25	-25.2	4.9

E10	34	-24.0	5.5	D01	26	-25.3	5.2
E10	35	-23.8	6.0	D01	27	-25.2	5.9
E10	36	-23.7	5.8	D01	28	-25.0	6.2
E10	37	-23.8	5.7	D01	29	-24.9	6.3
E10	38	-23.8	6.2	D01	30	-24.9	6.3
E10	39	-23.7	6.1	D01	31	-24.8	6.2
E10	40	-23.3	5.6	D01	32	-24.9	6.1
E10	41	-23.3	5.0	D01	33	-24.8	6.0
E10	42	-24.2	4.6	D01	34	-24.7	6.1
E10	43	-24.2	4.2	D01	35	-24.6	6.1
E10	44	-24.0	4.0	D01	36	-25.2	6.0
E10	45	-24.4	3.9	D01	37	-25.7	5.9
E10	46	-24.7	4.4	D01	38	-26.1	5.6
E10	47	-24.2	5.5	D01	39	-26.0	5.3
E10	48	-24.2	5.6	D01	40	-26.0	5.3
E10	49	-24.1	6.1	D01	41	-26.1	5.5
E10	50	-24.2	6.1	D01	42	-26.0	6.0
E10	51	-23.9	5.8	D12	0	-24.9	4.9
E10	52	-23.9	6.4	D12	1	-24.9	4.8
E10	53	-23.9	6.1	D12	2	-25.0	4.8
E10	54	-23.8	6.0	D12	3	-24.8	4.9
E10	55	-23.9	6.3	D12	4	-24.8	4.4
E10	56	-24.0	5.9	D12	5	-24.7	5.1
E10	57	-24.9	5.2	D12	6	-24.6	5.3
E10	58	-25.4	5.4	D12	7	-24.5	5.5
E10	59	-25.5	5.8	D12	8	-24.2	4.3
E10	60	-25.3	5.5	D12	9	-24.2	4.3
E10	61	-25.2	6.6	D12	10	-24.3	4.6
E10	62	-25.2	6.0	D12	11	-24.4	4.4
E10	63	-25.6	6.3	D12	12	-24.7	4.6
E12	0	-26.0	6.6	D12	13	-24.5	5.4
E12	1	-25.8	6.8	D12	14	-24.8	3.6
E12	2	-25.8	6.8	D12	15	-24.9	5.0
E12	3	-25.8	6.5	D12	16	-24.7	4.0
E12	4	-25.5	6.9	D12	17	-24.8	5.0
E12	5	-25.2	7.2	D12	18	-24.8	5.4
E12	6	-25.0	7.4	D12	19	-24.9	4.7
E12	7	-25.2	7.5	D12	20	-24.9	5.4
E12	8	-25.6	7.6	D12	21	-24.9	4.5
E12	9	-25.8	7.8	D12	22	-24.9	5.0
E12	10	-25.7	8.2	D12	23	-25.1	5.0
E12	11	-25.6	8.5	D12	24	-24.5	3.8
E12	12	-25.6	7.9	D12	25	-24.3	4.1
E12	13	-25.6	8.2	D12	26	-24.3	4.5

E12	14	-25.7	8.2
E12	15	-25.6	8.3
E12	16	-25.5	8.3
E12	17	-25.5	8.1
E12	18	-25.5	8.1
E12	19	-25.5	8.1
E12	20	-25.3	8.1
E12	21	-25.1	8.0
E12	22	-24.5	8.4
E12	23	-24.1	8.3
E12	24	-23.3	8.5
E12	25	-23.2	8.9
E12	26	-23.7	8.7
E12	27	-24.6	7.3
E12	28	-25.3	6.9
E12	29	-25.7	6.1
E12	30	-25.9	5.9
E12	31	-26.0	6.0
E12	32	-26.0	6.4
E12	33	-26.0	6.5
E12	34	-25.5	7.0
E12	35	-25.4	7.1
E12	36	-25.7	7.7
E12	37	-25.5	8.1
E12	38	-25.4	8.4
E12	39	-25.6	8.0
D06	0	-24.2	6.7
D06	1	-24.4	6.7
D06	2	-24.4	6.6
D06	3	-24.2	6.5

D12	27	-24.6	4.2
D12	28	-24.6	4.4
D12	29	-24.8	4.7
D12	30	-24.7	5.8
D12	31	-24.7	5.0
D12	32	-24.7	5.9
D12	33	-24.6	6.1
D12	34	-24.5	5.8
D12	35	-24.7	5.9
D12	36	-24.4	5.9
D12	37	-24.7	5.8
D12	38	-24.9	5.9
D12	39	-24.9	5.6
D12	40	-24.4	5.1
D12	41	-23.9	5.2
D12	42	-24.0	3.7
D12	43	-24.2	3.8
D12	44	-24.8	3.6
D12	45	-24.7	4.3
D12	46	-24.5	5.1
D12	47	-24.7	5.0
D12	48	-24.9	4.6
D12	49	-24.7	5.2
D12	50	-24.4	5.4
D12	51	-24.5	5.0
D12	52	-24.3	5.7
D12	53	-24.4	4.8
D12	54	-24.9	4.8
D12	55	-25.3	4.5
D12	56	-25.7	5.4
D12	57	-25.7	4.3
D12	58	-25.7	4.4
D12	59	-25.7	5.6
D12	60	-24.9	5.4
D12	61	-24.9	5.5
D12	62	-24.6	6.3

29
